# Novel approach to meniscal vascularity evaluation using indocyanine green fluorescence-guided knee arthroscopy

**DOI:** 10.1136/bmjsit-2024-000351

**Published:** 2025-06-25

**Authors:** Tamiko Kamimura

**Affiliations:** 1 Department of Orthopaedic Surgery, Tokorozawa Chuo Hospital, Tokorozawa, Saitama, Japan

**Keywords:** Arthroscopy, Health Technology, Technology Assessment, Biomedical, Development Study

## Abstract

**Objectives:**

This study aimed to use indocyanine green (ICG) fluorescence-guided knee arthroscopy to observe the meniscus and surrounding tissue vascularity and determine correlation with the patients’ backgrounds. Currently, no data are available on the clinical application of ICG fluorescence-guided knee arthroscopy in assessing meniscal vascularity.

**Design:**

Prospective, case series.

**Setting:**

In-hospital settings.

**Participants:**

41 knees of 34 patients were examined. 4 knees of 4 patients were included in a pilot study for technique refinement only, while the remaining 37 knees of 30 patients were included in the study.

**Main outcome measures:**

The times from ICG administration to fluorescence onset and fluorescence duration from onset to complete attenuation were recorded. The fluorescence intensity at the anterior, middle, and posterior segments of the meniscus was evaluated on a 4-point scale. The younger and older and smoker and non-smoker groups were compared.

**Results:**

The average fluorescence onset time was 32.05 s, whereas the average fluorescence duration was 11 min 14 s. The age groups aged≤45 and ≥46 years showed an onset of 30±24.9 and 33.17±16.2 s and a duration of 12 min 54 s and 10 min 20 s, respectively. The smoking and non-smoking groups exhibited an onset of 28.33±14.4 and 33.84±21.5 s and a duration of 10 min 37 s and 11 min 32 s, respectively. All segments of the lateral meniscus showed higher fluorescence intensities than the medial. The posterior segment of the lateral meniscus at ≤45 was markedly more fluorescent and significantly different from ≥46.

**Conclusions:**

Fluorescence was observed for approximately 30 s after intravenous ICG injection and lasted approximately 10 min. Fluorescence intensity was brighter in the posterior segment of the lateral meniscus, particularly at ≤45. ICG fluorescence-guided knee arthroscopy may assist in case-specific hemodynamics and real-time surgical evaluation of the meniscus in living humans.

**WHAT IS ALREADY KNOWN ON THIS TOPIC**

- Indocyanine green (ICG) is a cyanine dye widely used for hemodynamic evaluation mainly in the liver and heart. When near-infrared light is applied to tissue, ICG-containing tissue fluoresces; ICG-guided surgery is now being clinically applied in laparoscopy.

**WHAT THIS STUDY ADDS**

- The ICG fluorescence procedure enabled the real-time visualization of meniscal vascularity in individual patients. Fluorescence onset, duration, and intensity were recorded.

**HOW THIS STUDY MIGHT AFFECT RESEARCH, PRACTICE OR POLICY**

- Age, smoking, and other factors that generally affect blood flow and are poor prognostic factors for meniscal treatment may be better assessed using ICG fluorescence-guided knee arthroscopy, potentially aiding in individualized prognosis prediction.

## Introduction

 The meniscus significantly contributes to the primary function of the knee joint.

Its main functions are the distribution and transmission of load across the knee joint, acting as secondary stabilizers. It also provides proprioception for sensing joint position, which in turn prevents injuries. Meniscal injuries are commonly encountered in daily clinical practice and can result in decreased function, which can significantly accelerate the progression of knee osteoarthritis (OA).^1^

**Figure 1:**
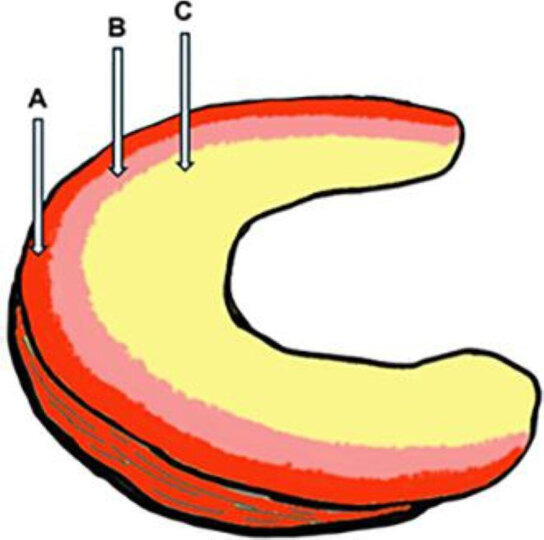
Schematic diagram showing vascularity in the meniscus. (A) Red–red zone: transition area of the meniscus and synovial capsule. (B) Red–white zone: the area between the red-red zone and the avascular zone. (C) White–white zone: avascular zone, considered extremely poor in vascularity.

Therefore, preserving the meniscus as much as possible to prevent future risk of OA represents a treatment goal in the field. However, the meniscus is anatomically poorly vascularized; the general thought is that vascularity is limited to the outer 10–30% of its volume. Detailed experimental results on poor blood supply to the meniscus were reported by King and Niebauer^2^ and Arnoczky and Warren.^3^ Advances in treatment of the meniscus developed after recognizing the red–red and red–white (vascular) and white–white (avascular) zones,^4^ starting from the meniscocapsular region as the outer synovial side of the meniscus (figure 1). Although it is commonly thought that injuries in the vascular zone could be repaired surgically due to the potential for healing, healing following meniscal repair, even in the vascular zone, is challenging. In the avascular zone, partial meniscectomy is recommended because of its poor healing potential. However, recent advances in biological augmentation have made healing of the avascular zone possible in some cases; it is thought that there is no need to leave the meniscus untreated owing to its limited vascularity.^5 6^ In clinical practice, partial meniscectomy has long been considered the gold standard for treating meniscal injuries. This procedure may provide temporary relief by removing the damaged meniscal region, with a shorter recovery time compared with meniscal repair; athletes can resume their sporting activities relatively soon. However, this procedure may also contribute to the development of OA in the long run.^1 7^ Even now, the uncertainty of meniscal repair due to poor vascularity of the meniscus makes many knee surgeons refrain from performing meniscal repair when they are unsure of whether to perform meniscectomy or meniscal repair. As a result, they give up on meniscal preservation. Due to the challenge in predicting the outcomes of meniscal repair, surgeons who prefer achieving clear prognoses often choose meniscectomy. However, the inability to visualize meniscal vascularity is apparently the underlying dilemma of this treatment.

The cyanine dye indocyanine green (ICG) is a well-established agent with a long history of use as a marker to assess organ blood flow with few side effects. This dye rapidly binds to the plasma proteins via intravenous injection; its characteristic fluorescence enhancement occurs under near-infrared (NIR) light with a wavelength of approximately 780–805 nm.^8^ It is one of the techniques used to identify anatomical structures and tissue vascularization for clarifying the boundaries between them.

In recent years, this characteristic has become a popular option for evaluating perfusion of target organs during laparoscopic surgeries.^9 10^ Real-time navigation for detecting vascularity contributes to improved safety, which is important for assessing case-specific organ perfusion during surgery.^11 12^ Surgical outcomes might improve owing to the availability of real-time information for intraoperative decision-making and predicting prognosis, such as anastomotic leakage. A substantial body of evidence demonstrates its effectiveness: in the field of general and laparoscopic surgeries, ICG fluorescence-guided surgery has been reported to improve intraoperative safety and contribute to improved postoperative outcomes.^12^,^14^

The author thought that intraoperatively laparoscopically assessing case-specific meniscal vascularity, which is key to the healing potential of the meniscus, could be useful for predicting the prognosis of meniscal surgery.

Since there have been no procedures to assess unique meniscal vascularity in living individuals, the author suggested that evaluating this vascularity using ICG fluorescence-guided knee arthroscopy might resolve these issues. This study aimed to evaluate the hemodynamics of the human meniscus using ICG fluorescence-guided knee arthroscopy to determine how the findings can be observed, how soon after ICG administration fluorescence is observed and where it is inferred that the meniscus has good vascularity, that is, where fluorescence is observed. Knowledge about the factors that have been proposed as poor prognostic factors for meniscal healing is extremely vague, with mixed opinions. In addition, data on the relationship between patient background and risk factors for meniscal repair are scarce, even in basic experiments. The author also aimed to evaluate the hemodynamics of the human meniscus using ICG fluorescence-guided knee arthroscopy in patients with different age and smoking backgrounds, factors that have been previously thought to influence meniscal healing, and determine whether the findings of this evaluation were related to the patients’ background.^15^,^18^

## Methods

This study represents the first clinical application of an ICG-based perfusion assessment technique, including its procedure, for visualizing intra-articular vascularity of the knee. Although the ICG fluorescence technique has been well established in laparoscopy, its application in knee arthroscopy is still novel. The primary focus of this study was to assess the feasibility, safety, and procedural optimization of this technique.

At this stage, efforts were directed towards optimizing and standardizing the technique in a limited number of cases. Therefore, this study corresponds to IDEAL Stage 2a. The study was designed as a prospective, single-center, single-surgeon (author) investigation. Furthermore, since intra-articular perfusion assessment using knee arthroscopy is a novel approach, a systematic evaluation of the technique was performed, with incorporating adjustments made as the technology evolved and more cases were included. The study commenced in September 2018.

### Patient and public involvement

The patients were individually informed about the established evidence supporting ICG fluorescence-guided laparoscopic surgery and its potential application in knee arthroscopy. Participation was entirely voluntary, and only those who acknowledged the potential contributions of this study to future surgical advancements chose to participate after receiving a comprehensive explanation about the study’s purpose and methodology.

### Patient selection

The cases in this study were those who presented to the author’s outpatient clinic for the treatment of meniscal injuries and opted to undergo meniscal repair for meniscal preservation. Postoperative patients with meniscal repair during the follow-up who chose to undergo repeat arthroscopy as a ‘second look’ for healing evaluation and/or suture implant removal were also included. Of these, patients with no history of allergy or diseases requiring long-term treatment were included. Patients who showed abnormal liver or kidney function during preoperative tests or a history of thyroid disorders were excluded from the study. Only eligible patients who understood the study’s purpose and provided informed consent were included in the study.

In total, 41 knees of 34 patients were included in this study. Among these, four knees from four patients were included in an initial pilot phase aimed at refining the fluorescence evaluation technique, during which no systematic data collection was performed. Consequently, the main study consisted of 37 knees of 30 patients.

### Anesthesia and surgical environment

Anesthesia and the surgical environment were standardized for managing all patients. General anesthesia was performed with an ultrasound-guided femoral nerve block and infiltration between the popliteal artery and the capsule of the knee in the affected limb.

A tourniquet was not used; hemostatic drugs were not administered.

An arthroscopic pump was used to ensure a uniform flow rate of 0.3 L/min and a pressure of 40 mm Hg to maintain a clear field of view. This was the lowest pressure and flow setting that could ensure clear arthroscopic vision and maintain as much natural blood flow in the affected limb as possible. Additionally, an irrigation trocar cannula, 2.0 mm in diameter, was placed for fluid outflow from the lateral suprapatellar region.

### Equipment for ICG fluorescence-guided knee arthroscopy

#### Camera system

**Figure 2:**
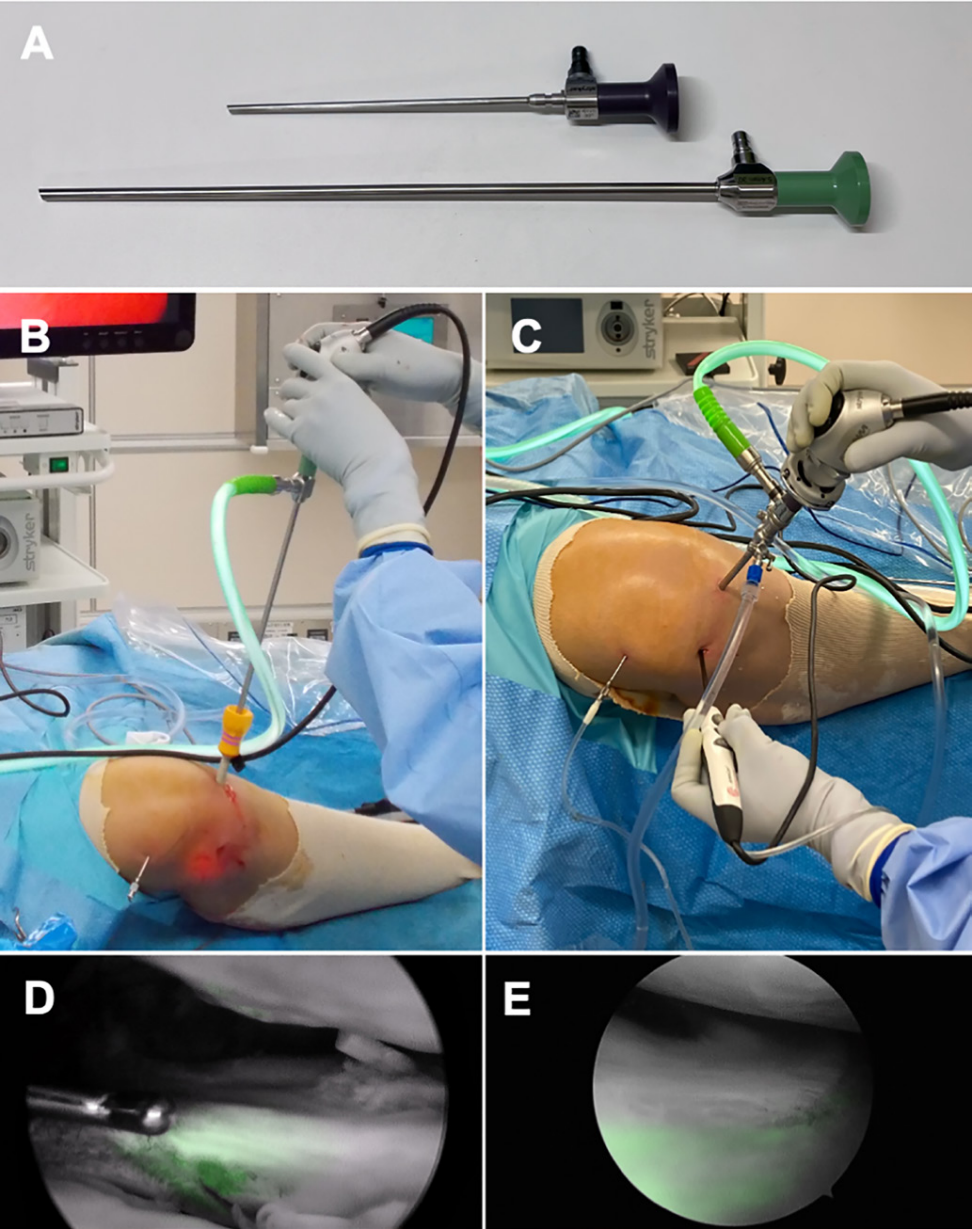
Comparison between laparoscope and arthroscope. (A) Side-by-side length comparison between the arthroscope (140 mm length, 4.0 mm in diameter, upper row) and laparoscope (300 mm length, 5.4 mm in diameter, lower row), clearly demonstrating the greater length of the laparoscope. Intraoperative photographs: (B) using a laparoscope and (C) using an arthroscope. Differences in the field of view: the posterior horn of the lateral meniscus is visualized with the laparoscope (D) and the anterior horn of the lateral meniscus is observed with the arthroscope (E). Note: For panels (B) and (C), the endoscope was inserted through the medial portal, rather than the lateral portal used in the standard protocol, to provide a clearer visual comparison of the scopes.

A Stryker’s 1588 camera combined with an L10 light source (Stryker, Kalamazoo, Michigan, USA) was used. Regarding ICG fluorescence-guided knee arthroscopy using these applications, the fluorescent green localization of ICG was reflected on a monochrome background (figures2  3). The camera system used by the author was designed to identify critical anatomical structures using advanced imaging modalities. The endoscopic near-infrared visualization (ENV) modality was used to visualize blood flow in real time, being capable of biliary mapping. Fluorescence-guided arthroscopy was used to observe two modality patterns: standard arthroscopy and the ENV mode.

**Figure 3:**
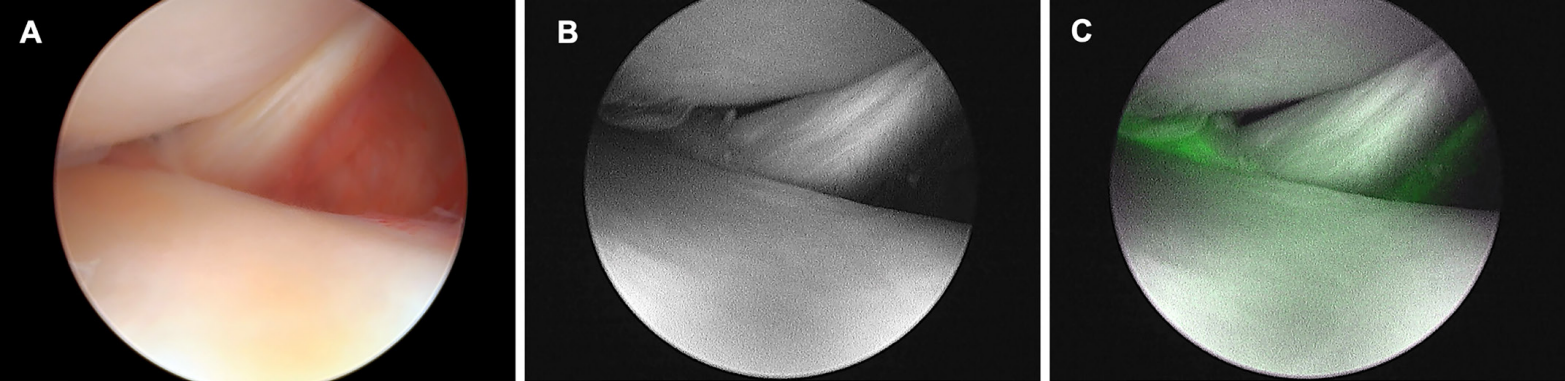
Sequence of protocols for detection of fluorescence under arthroscopy. (A) The arthroscope is positioned from the point of view of the popliteal tendon using the lateral infrapatellar approach in ‘Figure-4’ position. (B) To ensure this condition, the camera system is switched to the ICG (ENV) mode while maintaining the state in (A). (C) After waiting for about 30 s, fluorescence appears in this area after 2.0 mL of ICG solution (2.5 mg/mL) has been administered intravenously. ENV, endoscopic near-infrared visualization; ICG, indocyanine green.

**Figure 4:**
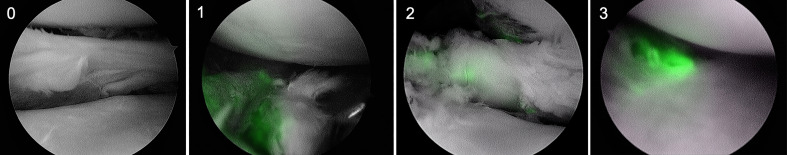
Observation of fluorescence brightness distribution: intensity evaluation levels. Level 0: no fluorescent. Level 1: no glowing green. Level 2: light fluorescent green. Level 3: bright fluorescence.

#### Arthroscopy pump

An arthroscopy pump (FloSteady Arthroscopy Pump, Stryker, Kalamazoo, Michigan, USA) was used to maintain a clear view without a tourniquet.

#### Scope

A pilot study was conducted including four cases without systematic data collection using a 5.4 mm laparoscope. Of the 37 main study cases, in the first 12, a 5.4 mm laparoscope was also used instead of a 4.0 mm arthroscope compatible with ICG fluorescence, as it was under development and had not yet been approved. The remaining 25 cases were examined using the approved arthroscope.

### Preparation of ICG solution for injection for this study protocol

ICG (Diagnogreen for injection, 25 mg; Daiichi Sankyo, Tokyo, Japan) was used for this study. ICG was in solid powder form; it was dissolved in 10 mL of sterile water to make a solution of 2.5 mg/mL for injection.

### Pilot study for technique refinement and modification

A pilot study of four cases was conducted using an iterative process to optimize the observation protocol for ICG fluorescence-guided knee arthroscopy. In this phase, both ICG dosages of 2.0 and 2.5 mL at 2.5 mg/mL concentration were tested based on previous clinical reports. Fluorescence intensity, visualization consistency, and procedural feasibility were evaluated to determine the optimal protocol. These findings informed the development of the protocol for the subsequent 37 cases. No systematic data were collected, as the goal was to refine the protocol.

The pilot study demonstrated that both 2.5 and 2.0 mL of ICG at a concentration of 2.5 mg/mL resulted in similar fluorescence visualization. To establish a standardized protocol, the author adopted 2.0 mL as the uniform dose for the main study, considering it sufficient for imaging quality while minimizing dye usage.

During the course of this study, the author conducted follow-up animal experiments and confirmed that this dose was optimal.^19^ Additionally, the pilot study findings also suggested that a low ENV gain setting was essential for reducing halation and improving visibility. At high ENV gain, halation appeared even without ICG administration; the coloration of intra-articular structures became overly intense with ICG, resulting in poor contrast. By reducing the gain, contrast was enhanced, allowing clearer visualization of joint structures with actual ICG fluorescent vascularity. The ENV mode provides five gain options. Based on the pilot study findings, the author initially set the ENV gain to level 2—the second-lowest setting—for the subsequent 37 cases in the main study. If halation interfered with observation, the gain was further reduced to level 1 to improve visibility.

Two different scopes were used throughout the pilot and main study. In the initial cases, including the pilot study, a laparoscope was used because an arthroscope for ICG fluorescence was still under development and not yet available. The laparoscope is longer, less maneuverable, and has a wider angle of view (figure 2).

Since the laparoscope is challenging to handle in knee arthroscopy, video assessment was adopted to enhance fluorescence evaluation. While fluorescence was assessed intraoperatively, a subsequent video review was conducted for verification.

The observation sequence was refined during the pilot study phase. In contrast, the ICG-compatible arthroscope made available during the mid-stage of this study offered improved operability, allowing for simultaneous assessment in all except the first three, where both video and simultaneous assessment were combined. All the procedures were fully recorded to ensure an accurate and consistent assessment. The progression of modifications is outlined in table 1.

**Table 1:** Phases and modifications summary

Patient No.(Study #)	Phase	Cases, n	ICG dosage(2.5 mg/mL)	Fluorescence evaluation	Modifications
1–4(Pilot)	Phase 0Pilot(laparoscope)	4	2.0 and/or 2.5 mL	Investigation of technique and assessment of dosage	Tested both 2.0 and 2.5 mL ENV gain level adjustment No observable difference between dosages and fluorescence
5–16(1–12)	Phase I(laparoscope)	12	2.0 mL	Video review adopted	Adoption of video assessment to address uncertainty in fluorescence evaluation Improved consistency
17–19(13–15)	Phase II(arthroscope: initial 3 cases)	3	2.0 mL	Video and simultaneous assessment adopted	Improved handling with arthroscope Transition from laparoscope
20–41(16–37)	Phase III(arthroscope)	22	2.0 mL	Simultaneous assessment adopted	Improved operability of arthroscope Simultaneous assessment adopted

ENV, endoscopic near-infrared visualization; ICG, indocyanine green.

### Study population and characteristics of participants

A total of 37 knees of 30 patients were included in the main study. Seven patients (14 knees) underwent repeat arthroscopy as a second-look procedure. The participants were 13 male and 17 female patients with a mean age at surgery of 45.1±17.9 (13–76) years. Patient demographics and medical histories are listed in table 2.

**Table 2:** Characteristics of participants

 Characteristics	Total 37 knees in 30 patients
Age (at surgery), years	45.1±17.9 (13–76)
Sex	Male: 13, female: 17
BMI (kg/m^2^)	23.6±2.8
Affected knee	Right: 20Left: 17
Primary procedureor second look, n	Primary: 23Second look or revision: 14
Meniscal tear, n	Medial: 13Lateral: 22Medial and lateral: 2
Smoking status, n	Yes: 12No: 25
History of present illness, n	Hypertension: 3, hyperlipidemia: 1,sleep apnea: 1, ulcerative colitis: 1,gastroesophageal reflux disease: 1
History of past illness, n	Varicose veins: 1, cholecystolithiasis: 1
HbA1c (at surgery)	5.6±0.36 (5.0–6.4)

BMI, body mass index; HbA1c, hemoglobin A1C.

### The examination protocol of ICG fluorescence-guided knee arthroscopy

ICG fluorescence-guided knee arthroscopy was performed in all cases in a non-invasive fashion prior to the intended surgery. Prior to ICG administration, routine diagnostic arthroscopy was performed using the standard lateral and medial infrapatellar approaches as the usual portal, with minimal probing and without manipulation that would affect bleeding or blood flow.

The arthroscope was positioned at the point of view of the popliteal tendon using the lateral infrapatellar approach. The patient’s posture at this point was in ‘Figure-4’ position. To ensure this condition, the camera system was switched into the ICG (ENV) mode, and the ENV gain was initially set to level 2 in this investigation, examining all cases. In cases where the halation effect was excessively pronounced, the gain was adjusted downward to level 1.

Subsequently, 2.0 mL of ICG solution (2.5 mg/mL) was injected intravenously, followed by 20 mL of saline and left until fluorescence could be visualized in this state (figure 3).

### Fluorescence onset and duration

The time at which fluorescence was observed in this area (the popliteal region of the lateral meniscus) was recorded as the ‘onset’ time. Observations were made starting with the posterior segment of the lateral meniscus, followed by the middle and anterior segments, moving to the medial meniscus by extending the knee and then the anterior, middle, and posterior segments. The time of complete attenuation of fluorescence within the joint was recorded as ‘the end’. The ‘duration’ of the fluorescence observation time was calculated as the time between the end and the onset of the observation.

### Observation of fluorescence brightness distribution

The author observed and recorded where the fluorescence was distributed and the degree of brightness that could be observed under arthroscopy. The fluorescence intensity at the posterior, middle, and anterior segments of the lateral and medial menisci was evaluated on a scale of 0–3. The evaluation levels are shown in figure 4: level 0 (no fluorescence), level 1 (no glowing green), level 2 (light fluorescent green), and level 3 (bright fluorescence).

The results were also analyzed with respect to age and smoking status, which have been reported to influence healing after meniscal repair. The author also evaluated the patient groups divided by the mean age≤45 and ≥46 years and by smokers and non-smokers, respectively.

### Statistical analyses

Statistical analyses were conducted using the R software (R Foundation for Statistical Computing, Vienna, Austria). Graphical representations were generated using Microsoft Excel (Microsoft Corporation, Redmond, Washington, USA).

## Results

### Onset and duration of the fluorescence

**Figure 5:**
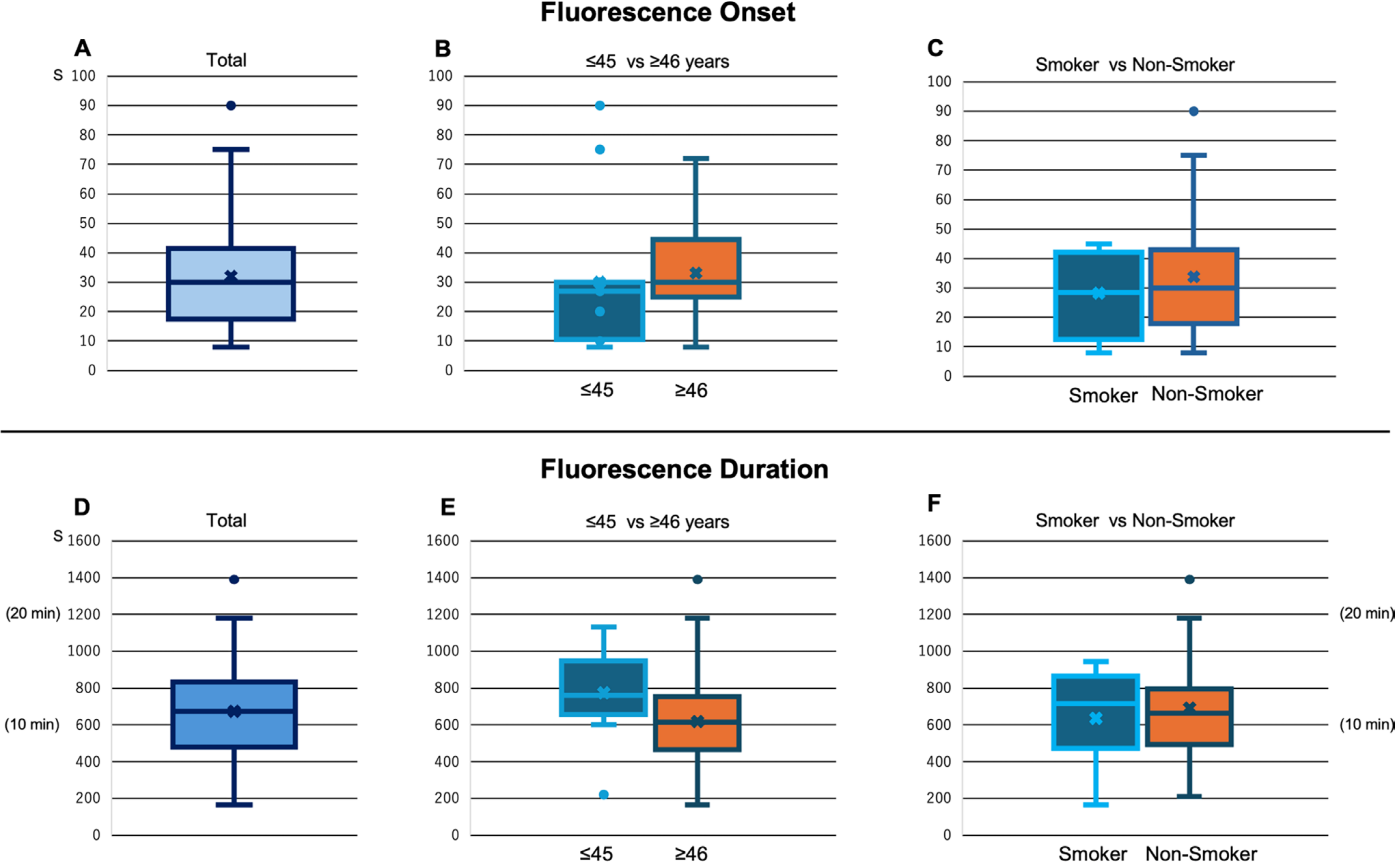
Fluorescence onset and duration in each group. (A) The fluorescence onset time results in all cases; the average onset time is 32.05 s. (B) Comparison of onset in ≤45 and ≥46 years. (C) Comparison of onset in smokers and non-smokers. (D) The fluorescence duration time results in all cases; the average duration is 11 min and 14 s. (E) Comparison of duration in ≤45 and ≥46 years. (F) Comparison of duration in smokers and non-smokers.

The average time for fluorescence onset was 32.05 s, whereas the average fluorescence duration was 11 min and 14 s (figure 5). The earliest onset was 8 s and the slowest was 90 s. The earliest, 8 s, onset occurred in two cases: a patient in their 20s undergoing a second look after discoid lateral meniscus surgery and a patient in their 40s with a discoid lateral meniscus. These two cases were parent and child. The slowest occurred in a case of a patient in their 20s without a history of smoking. Regarding duration, the shortest and longest were only 2 min and 45 s and 23 min and 10 s, respectively. The cases exhibiting the shortest and longest duration were a patient in their 50s with a long history of smoking and a patient in their 70s with a history of multiple varicose vein surgeries within 2 years, respectively.

The patients were allocated into two groups by mean age: those aged≤45 and ≥46 years, showing an onset of 30±24.9 and 33.17±16.2 s, respectively; there was no significant difference (p=0.643). The duration in these two groups was 12 min 54 s (774.3±236.6 s) and 10 min 20 s (619.5±290.2 s), respectively, without showing a statistically significant difference (p=0.108). The smoking and non-smoking groups exhibited an onset of 28.33±14.4 and 33.84±21.5 s, respectively; there was no significant difference (p=0.428). The duration in these groups was 10 min 37 s (636.7±257 s) and 11 min 32 s (691.7±293 s), respectively, without showing a statistically significant difference (p=0.582) (figure 5). Among the groups divided by age, the ≤45 years group showed a tendency towards early onset and long duration, whereas the ≥46 years group showed late onset and short duration. Among the groups of smokers and non-smokers, the average onset tended to be earlier, and duration was shorter among the smokers.

### Locations where fluorescence was observed in the meniscus

**Figure 6:**
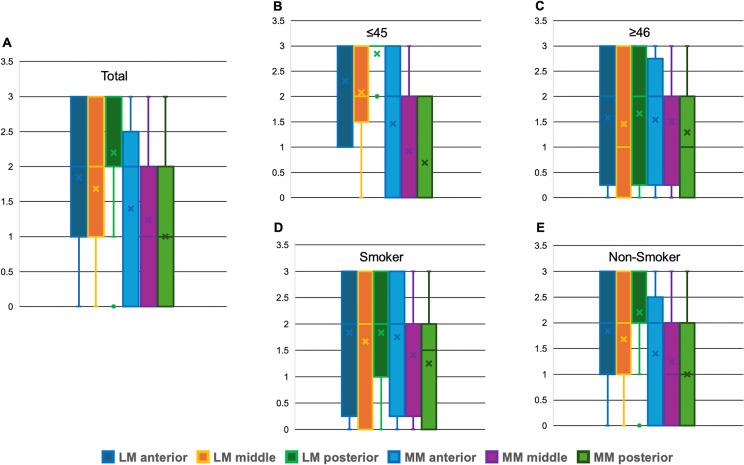
Fluorescence location and intensity levels in each group. (A) Fluorescence intensity levels in all cases. All segments of the lateral meniscus show higher fluorescence intensities than do those of the medial meniscus. (B,C) In the two groups divided by mean age, all sites show higher fluorescence at age≤45 years and lower fluorescence at age≥46 years in the lateral meniscus. (D,E) Among smokers and non-smokers, smokers exhibit lower intensity levels, particularly in the lateral meniscus. LM, lateral meniscus; MM, medial meniscus.

Fluorescence was observed only in the vascular area, as indicated by the red–red and red–white zones. Although it depended on the case, the posterior, middle, and anterior segments of the lateral and medial menisci examined in this study all showed fluorescence. The results of the level of fluorescence brightness are shown in figure 6. The fluorescence intensity was higher in the lateral meniscus than that in the medial meniscus. In the site-specific evaluation of each meniscus, the posterior segment of the lateral meniscus was observed to be particularly fluorescent, whereas the posterior segment of the medial meniscus was observed to be the least fluorescent.

In the two groups divided by mean age, all sites in the lateral meniscus showed higher fluorescence at age≤45 years and lower fluorescence at age≥46 years. Comparing these two groups using the Mann-Whitney U test, only the posterior segment of the lateral meniscus was significantly different (p=0.002). No significant differences were found at the other sites of the lateral and medial meniscus; in the lateral meniscus: the anterior (p=0.064) and middle (p=1.425) segments and in the medial meniscus: the anterior (p=0.908), middle (p=0.135), and posterior (p=0.101) segments. However, regarding the medial meniscus, there was rather a trend towards lower fluorescence in the ≤45 years group.

Compared with smokers and non-smokers, the smokers exhibited lower intensity levels in the lateral meniscus. Considering the medial meniscus, the non-smoker group tended to have lower fluorescence intensity, inversely related to the lateral meniscus. However, there were no significant differences between these groups at any sites of the lateral and medial meniscus; in the lateral meniscus: the anterior (p=1.000), middle (p=0.960), and posterior (p=0.360) segments and in the medial meniscus: the anterior (p=0.399), middle (p=0.837), and posterior (p=0.585) segments.

The results showed similar trends in the lateral meniscus fluorescence between the group aged≤45 years, non-smokers, and the group aged≥46 years, and smokers (figure 6).

## Discussion

The most important finding in this study was the elucidation of the fundamental observations during ICG fluorescence-guided knee arthroscopy for assessing meniscal vascularity. This study focused on key aspects: measuring the time taken until fluorescence became detectable, measuring how long fluorescence remained visible during arthroscopy, identifying specific meniscal areas with notable fluorescence, and assessing relationships with patient characteristics such as age and smoking habits. These findings could play a crucial role in developing standardized guidelines for interpreting ICG uptake patterns in the meniscus during future arthroscopic procedures.

Blood supply to the meniscus is known to be extremely poor; this is based on the study by Arnoczky and Warren.^3^ They revealed that blood supply to the human meniscus at its edge and the area from the synovium to one-third of the meniscus is known as the red zone, and the intercondylar side is termed as the white zone (figure 1). This division into red and white zones is widely used for segmental classification of red-to-white zones^4^; even presently, it is thought that only meniscal injuries in the red zone can heal, and the location of the meniscal injury is the only key to determining the suitability of meniscal repair and predicting prognosis.

However, recent experimental studies have reported the absence of blood vessels in the vascular zone,^16^ as well as the presence of blood vessels in the avascular zone.^20 21^ As a result, data on meniscal vascularization are still unclear. An additional challenge in meniscal treatment is the unpredictable nature of the healing process. Even in the red–red zone, which is thought to have an adequate blood supply, healing of this site is not guaranteed even if biological augmentation is added. Conversely, areas presumed to lack vascularity may unexpectedly heal better than anticipated, further complicating clinical management.^22^

These raise some questions about management strategies. Moreover, the prognostic prediction of meniscal repair based on the classification of red-to-white zones is limited as an indicator. The author hypothesized that if vascularity of the meniscus could be visualized on an individual basis, as a case-specific evaluation, it could help bridge the gap between past and current research findings, ultimately contributing to the establishment of intraoperative strategies and helping predict prognosis. This fundamental concept serves as the basis for applying ICG to knee arthroscopy.

The development of innovative surgical instruments and the expertise of surgeons have played a significant role in the remarkable progress made in ICG fluorescence-guided surgery. The first use of ICG fluorescence-guided cholecystectomy was reported by Ishizawa *et al*,^23^ which garnered growing interest in optical imaging guidance techniques that employ endoscopy with an NIR light source to accurately assess the target organ.^9^,^1224^ The NIR-ICG fluorescence technique has made remarkable progress in the surgical field, playing an important role not only in ensuring greater surgical safety^11 13^ but also in checking the effectiveness of cancer treatment^25 26^ and lymph node mapping.^26^,^28^ Furthermore, tissue perfusion assessment indicating its effectiveness^29 30^ and its quantification is enabling prognostic prediction.^31^,^34^ Although ICG fluorescence-guided surgery has limited application in the field of orthopedics, clinical studies have been reported in recent years on shoulder arthroscopy. Doi *et al*^35^ and Shibata *et al*^36^ examined the blood flow of the rotator cuff using ICG fluorescence-guided shoulder arthroscopy and described the relationship between intraoperative findings and postoperative results of rotator cuff tears.

Clinical applications of ICG fluorescence-guided surgery have primarily focused on organs with good blood flow to evaluate areas with insufficient and disease-specific blood flow. However, with the exception of the synovium, there is little soft tissue in the knee joint, and its blood flow is also sparse. Therefore, the results obtained from the knee joint are likely to differ from those of previous clinical applications and will be used to identify areas with good blood flow, with a particular emphasis on regions exhibiting poor perfusion.

In this study, the author found that ICG fluoresced approximately 30 s after administration and lasted for 10 min before attenuation. The factors that influence this include the speed of administration and size of the route of vascularization; intraoperative position; size of the thigh muscle group; blood pressure; arterial stiffness; and joint degeneration. Since the fluorescence duration in the knee joint was as short as 10 min, effective observation within this limited timeframe would be required.

Aging and smoking have also been reported to affect meniscal healing.^15^,^18^ However, some argue that neither age nor smoking plays a role in meniscal healing outcomes.^37 38^ Furthermore, a microscopic examination of degenerative menisci reported that neovascularization was actually observed in smokers.^39^ It remains to be determined whether age, smoking, and even atherosclerosis, which are known to impair tissue perfusion, might influence meniscal healing.

Although the analysis of age and smoking status in this study was not conclusive, a certain trend emerged. Intriguingly, while exploring different patient backgrounds, the author observed rare cases of discoid lateral meniscus in a parent and child who exhibited the earliest onset of fluorescence, as well as a case with the longest fluorescence duration in a patient with a history of multiple varicose vein surgeries. The challenge lies in accumulating more cases and systematically examining the ICG uptake patterns in individual patients. Since ICG is originally used for evaluating blood flow, future evaluation methods may enable the examination of the relationship between factors such as aging, smoking, atherosclerosis, and meniscal healing potential, ultimately leading to the identification of additional factors influencing ICG uptake patterns and improving meniscal surgery outcomes. In this context, the author used a uniform ICG dosage in all main study cases to enable effective comparison of results. In the future, considering patient-specific factors, such as age, body height, weight, and relevant medical history of blood perfusion might be necessary, to improve the reliability and validity of the findings.

The author has observed some important findings on ICG fluorescence-guided knee arthroscopy. For example, areas where blood vessels were visible using the standard mode did not necessarily show fluorescence, even in the vicinity of beating blood vessels. A previous fundamental study indicated that the knee joint experiences constant loading, suggesting that blood vessels within the meniscus may be retarded or congested to distribute this load.^40^ According to this theory, the absence of fluorescence in the meniscus can be attributed to a reduction in vascularity resulting from the applied pressure. Accumulating more cases in the future might help further elucidate this point.

The study has limitations regarding the small sample size and group composition, with an uneven distribution of smokers and non-smokers and a higher frequency of affected areas in the lateral meniscus, which may have introduced selection bias. As this study was the initial investigation to assess meniscal vascularity using ICG fluorescence during knee arthroscopy, no pre-existing evaluation standards were available. Consequently, the author’s assessment might be subjective, and standardized evaluation methods have yet to be established. Additional clinical research employing uniform scoring systems and objective measurements would be valuable. Furthermore, investigating dose-dependent fluorescence intensity and dosing based on patient background to assess whether the current ICG dosing is indeed appropriate would be beneficial in future studies.

Furthermore, challenges in operability may partly stem from the camera system used in this study. Owing to the lack of an ICG-compatible arthroscope at the start of the study, a laparoscope was used, requiring confirmation through video assessment due to its limited maneuverability. The subsequent introduction of an ICG-compatible arthroscope enabled real-time assessment, significantly improving accuracy and efficiency. Moreover, the narrow space of the knee joint poses difficulties in maintaining an optimal distance for fluorescence visualization between the scope and target. Differences in spatial optimization between arthroscopes and laparoscopes may have led to variations in aberration correction, potentially introducing an optical bias that should be addressed in future studies. In addition, challenges remain in maintaining a clear field of view during knee arthroscopy with Ringer’s or saline solution, requiring ENV gain adjustments on the Stryker 1588 camera used in this study to reduce halos and distortions. Simultaneous surgery while visualizing blood flow was also difficult with the Stryker 1588 due to the monochrome display of ICG fluorescence. The Stryker 1688 camera system, which became available after initiating this study and supported real-time ICG fluorescence as an overlay on a standard white-light image, enabled surgery while visualizing blood flow. Using this system, the author confirmed that blood flow within the meniscus could be visualized by stimulating the synovium in an experimental pig model^19^ and that fluorescence intensity was easier to evaluate during human meniscal repair.^41^

Recent technological advancements have resolved issues beyond the capabilities of the Stryker 1688. The color mapping function of the Stryker 1788 camera, now available, may enable a more objective evaluation of meniscal blood flow. Additionally, the normalization feature of the Stryker 1788, which maintains a constant fluorescence intensity regardless of distance, could address one of the challenges identified in this study. The results of this study indicated that fluorescent observation during knee arthroscopy was limited to a short period. Therefore, developing a system that could simultaneously assist in visualizing and quantifying fluorescence is required.

Moreover, since interpreting the results might be challenging, the definite meaning of onset and duration of fluorescence should be further elucidated.

Similarly, the single-center, single-surgeon (author) design of this study may limit the interpretation of the results, affecting its reproducibility and generalizability. Further validation through multicenter studies involving multiple surgeons is warranted.

The greatest advantage of ICG fluorescence-guided knee arthroscopy is that the blood supply in the human meniscus, which has only been verified in cadaveric knees, could be evaluated intraoperatively in real time to assess the condition of the meniscus.

No complications were observed in this study; therefore, ICG could be safely applied to knee arthroscopy for patient evaluation, suggesting its potential to explore how patient background influences meniscal hemodynamics.

As case-specific vascularization is likely to be the key to meniscal healing, enhancing this technique in clinical settings could potentially reduce errors in managing meniscal injuries. This improvement might lead to a substantial decrease in the number of meniscectomies performed when meniscal repair is a viable option. It could also help avoid unnecessary meniscal repair in cases wherein healing is not feasible. To achieve these objectives, improving fluorescence quantification, establishing standardized scoring systems, and accumulating more clinical cases to enhance clinical applicability are essential. By addressing existing limitations, the accuracy and reliability of fluorescence-guided evaluation could be greatly improved.

The author hopes that ICG fluorescence-guided knee arthroscopy would enable better prediction of healing potential and surgical prognosis, addressing the limits of the red-to-white zone classification in future clinical practice. The main goal of this technique is to support intraoperative decision-making between meniscal repair and meniscectomy by assessing real-time vascularity of the injured meniscus. This approach, which allows for the direct assessment of meniscal vascularity in living humans, has the potential to improve surgical outcomes and contribute to preserving meniscal function, thereby improving patient quality of life.

## Conclusion

A clinical application of knee arthroscopy using an ICG fluorescence-guided procedure to evaluate meniscal vascularity was performed. Although many challenges were encountered, the results suggest that this technique may serve as a novel approach to investigate meniscal vascularity, which has not been confirmed until now, as a bio-optical navigation arthroscopy. This approach may assist in assessing case-specific hemodynamics and provide real-time surgical evaluation of the meniscus in living humans.

## Data Availability

All data relevant to the study are included in the article or uploaded as online supplemental information.

## References

[1] Skinner M, Sullivan B, Conley C (2024). Incidence of Osteoarthritis Diagnosis Within 5 Years of Surgery Was Greater Following Partial Meniscectomy Than Meniscus Repair and/or Anterior Cruciate Ligament Reconstruction. Arthrosc Sports Med Rehabil.

[2] King D, Niebauer JJ (1990). The healing of semilunar cartilages. 1936. Clin Orthop Relat Res.

[3] Arnoczky SP, Warren RF (1982). Microvasculature of the human meniscus. Am J Sports Med.

[4] Cooper DE, Arnoczky SP, Warren RF (1990). Arthroscopic meniscal repair. Clin Sports Med.

[5] Kamimura T, Kimura M (2014). Meniscal Repair of Degenerative Horizontal Cleavage Tears Using Fibrin Clots: Clinical and Arthroscopic Outcomes in 10 Cases. Orthop J Sports Med.

[6] Hashimoto Y, Nishino K, Orita K (2022). Biochemical Characteristics and Clinical Result of Bone Marrow-Derived Fibrin Clot for Repair of Isolated Meniscal Injury in the Avascular Zone. Arthroscopy.

[7] Abram SGF, Judge A, Beard DJ (2019). Long-term rates of knee arthroplasty in a cohort of 834 393 patients with a history of arthroscopic partial meniscectomy. Bone Joint J.

[8] Landsman ML, Kwant G, Mook GA (1976). Light-absorbing properties, stability, and spectral stabilization of indocyanine green. J Appl Physiol.

[9] Ahn H-M, Son GM, Lee IY (2021). Optimization of indocyanine green angiography for colon perfusion during laparoscopic colorectal surgery. Colorectal Dis.

[10] Terasawa M, Ishizawa T, Mise Y (2017). Applications of fusion-fluorescence imaging using indocyanine green in laparoscopic hepatectomy. Surg Endosc.

[11] Breuking EA, van Varsseveld OC, Harms M (2023). Safety and Feasibility of Indocyanine Green Fluorescence Angiography in Pediatric Gastrointestinal Surgery: A Systematic Review. J Pediatr Surg.

[12] Dip F, Boni L, Bouvet M (2022). Consensus Conference Statement on the General Use of Near-infrared Fluorescence Imaging and Indocyanine Green Guided Surgery: Results of a Modified Delphi Study. Ann Surg.

[13] Watanabe J, Ishibe A, Suwa Y (2020). Indocyanine green fluorescence imaging to reduce the risk of anastomotic leakage in laparoscopic low anterior resection for rectal cancer: a propensity score-matched cohort study. Surg Endosc.

[14] Gijsen AF, de Vries RPH, Vaassen HGM (2024). The use of indocyanine green fluorescence imaging in preventing postoperative bile leakage of the hepaticojejunostomy in robot-assisted pancreatic surgery. HPB (Oxford).

[15] Husen M, Kennedy NI, Till S (2022). Benefits of Meniscal Repair in Selected Patients Aged 60 Years and Older. Orthop J Sports Med.

[16] Michel PA, Domnick CJ, Raschke MJ (2021). Age-Related Changes in the Microvascular Density of the Human Meniscus. Am J Sports Med.

[17] Blackwell R, Schmitt LC, Flanigan DC (2016). Smoking increases the risk of early meniscus repair failure. Knee Surg Sports Traumatol Arthrosc.

[18] Uzun E, Misir A, Kizkapan TB (2017). Factors Affecting the Outcomes of Arthroscopically Repaired Traumatic Vertical Longitudinal Medial Meniscal Tears. Orthop J Sports Med.

[19] Kamimura T (2024). Blood Flow in the Meniscus Can Be Visualized Arthroscopically Using an Intravenous Indocyanine Green Solution Diluted 10× in a Pig Model. *Arthrosc Sports Med Rehabil*.

[20] Chahla J, Papalamprou A, Chan V (2021). Assessing the Resident Progenitor Cell Population and the Vascularity of the Adult Human Meniscus. Arthroscopy.

[21] Crawford MD, Hellwinkel JE, Aman Z (2020). Microvascular Anatomy and Intrinsic Gene Expression of Menisci From Young Adults. Am J Sports Med.

[22] Barber-Westin SD, Noyes FR (2014). Clinical healing rates of meniscus repairs of tears in the central-third (red-white) zone. Arthroscopy.

[23] Ishizawa T, Bandai Y, Kokudo N (2009). Fluorescent cholangiography using indocyanine green for laparoscopic cholecystectomy: an initial experience. Arch Surg.

[24] Barabino G, Porcheron J, Cottier M (2016). Improving Surgical Resection of Metastatic Liver Tumors With Near-Infrared Optical-Guided Fluorescence Imaging. Surg Innov.

[25] Ishizawa T, Masuda K, Urano Y (2014). Mechanistic background and clinical applications of indocyanine green fluorescence imaging of hepatocellular carcinoma. Ann Surg Oncol.

[26] Kusano M, Tajima Y, Yamazaki K (2008). Sentinel node mapping guided by indocyanine green fluorescence imaging: a new method for sentinel node navigation surgery in gastrointestinal cancer. Dig Surg.

[27] Watanabe J, Ohya H, Sakai J (2023). Long-term outcomes of indocyanine green fluorescence imaging-guided laparoscopic lateral pelvic lymph node dissection for clinical stage II/III middle-lower rectal cancer: a propensity score-matched cohort study. Tech Coloproctol.

[28] Aoki Y, Kanao H, Fusegi A (2022). Indocyanine green-guided sentinel lymph node mapping during laparoscopic surgery with vaginal cuff closure but no uterine manipulator for cervical cancer. Int J Clin Oncol.

[29] Li K, Zhang Z, Nicoli F (2018). Application of Indocyanine Green in Flap Surgery: A Systematic Review. J Reconstr Microsurg.

[30] Schols RM, Dip F, Lo Menzo E (2022). Delphi survey of intercontinental experts to identify areas of consensus on the use of indocyanine green angiography for tissue perfusion assessment during plastic and reconstructive surgery. Surgery.

[31] Abdelwahab M, Spataro EA, Kandathil CK (2019). Neovascularization Perfusion of Melolabial Flaps Using Intraoperative Indocyanine Green Angiography. JAMA Facial Plast Surg.

[32] Alstrup T, Christensen BO, Damsgaard TE (2018). ICG angiography in immediate and delayed autologous breast reconstructions: peroperative evaluation and postoperative outcomes. J Plast Surg Hand Surg.

[33] Van den Hoven P, S Weller F, Van De Bent M (2022). Near-infrared fluorescence imaging with indocyanine green for quantification of changes in tissue perfusion following revascularization. Vascular.

[34] Colvard B, Itoga NK, Hitchner E (2016). SPY technology as an adjunctive measure for lower extremity perfusion. J Vasc Surg.

[35] Doi N, Izaki T, Miyake S (2019). Intraoperative evaluation of blood flow for soft tissues in orthopaedic surgery using indocyanine green fluorescence angiography: A pilot study. Bone Joint Res.

[36] Shibata T, Doi N, Shibata Y (2024). Application of indocyanine green fluorescence angiography in evaluating blood flow in rotator cuff tears: a preliminary study. J Shoulder Elbow Surg.

[37] Rothermel SD, Smuin D, Dhawan A (2018). Are Outcomes After Meniscal Repair Age Dependent? A Systematic Review. Arthroscopy.

[38] Zabrzyński J, Paczesny Ł, Zabrzyńska A (2022). Smoking Has No Influence on Outcomes after Repair of the Medial Meniscus in the Hypo and Avascular Zones-A Pilot Study. Int J Environ Res Public Health.

[39] Zabrzyńska M, Pasiński M, Gagat M (2024). The Association between the Extent of the Osteoarthritic Meniscus Degeneration and Cigarette Smoking-A Pilot Study. Medicina (Kaunas).

[40] Gray JC (1999). Neural and vascular anatomy of the menisci of the human knee. J Orthop Sports Phys Ther.

[41] Kamimura T (2024). Indocyanine Green Fluorescence-Guided Knee Arthroscopy: A Technical Note for Investigating the Microvasculature Around the Meniscus. Arthrosc Tech.

